# A crossover study on attentional focus and gross motor performance in individuals with Down syndrome

**DOI:** 10.1371/journal.pone.0305267

**Published:** 2024-08-22

**Authors:** Luca Cavaggioni, Luca Paolo Ardigò, Paolo Castiglioni, Athos Trecroci, Linda Casalini, Damiano Formenti, Giampiero Merati

**Affiliations:** 1 Department of Endocrine and Metabolic Diseases, Obesity Unit—Laboratory of Nutrition and Obesity Research, IRCCS Istituto Auxologico Italiano, Milan, Italy; 2 Department of Biotechnology and Life Sciences, University of Insubria, Varese, Italy; 3 Department of Teacher Education, NLA University College, Oslo, Norway; 4 IRCCS Fondazione don Carlo Gnocchi, Milan, Italy; 5 Department of Biomedical Sciences for Health, Università degli Studi di Milano, Milano, Italy; 6 Italian Federation of Intellectual and Relational Disability Sports (FISDIR), Rome, Italy; Faculty of Health Sciences, University of Primorska, SLOVENIA

## Abstract

Little is known about the effect of using an attentional focus instruction on motor performance in people with intellectual disabilities. Therefore, this study explored the effects of different attentional focus instructions on gross motor skill performances in individuals with Down syndrome. Seven community-dwelling participants (age 25.2±3.2 yrs, height 1.70±0.04 m, body mass 72.0±6.3 kg) voluntarily participated in the study. Motor performance on 5-meter running (5m sprint), vertical jump (countermovement jump with arm swing, CMJ), broad jump (standing broad jump, SBJ), forward medball throw (FMBT) or overhead medball backward throw (OMBT) and rising-up from a chair (five repetition sit-to-stand, 5STS) were recorded while performing internal-focus (IF) or external-focus (EF) instructions. EF induced significantly (p<0.05) better performance than IF in CMJ (EF: 15±9 cm; IF: 11±8 cm, median ±interquartile range), SBJ (EF: 0.8±1.05 m; IF: 0.5±1.0 m), FMBT (EF: 1.5±1.4 m; IF: 1.4±1.1 m), OMBT (EF: 4.0±1.5 m; IF: 3.6±1.1 m) and 5STS (EF: 14.2±5.4; IF:15.3±7.7 s). The time over the 5m sprint tended to be shorter with EF (4.0±2.0 s) than IF (5.05±3.3 s) but the difference did not reach the statistical significance (p = 0.29). Physical trainers and school teachers should be encouraged to manage different types of attentional focus instructions to improve cognitive and gross motor performances in persons with Down syndrome.

## Introduction

The role of attentional focus concerning health or sports performance is multifaceted and has garnered significant consideration in various domains [1–3]. Research has shown that a verbal cue during training can shift the attention inside or outside the body (i.e., internal focus or external focus), provoking different consequences on both motor action and the learning process [4]. An internal focus refers to concentrating on one’s body movements, whereas an external focus means directing attention toward the external environment or the outcome of a movement. Internal focus supporters argue that by directing attention to the body, one gains a deeper understanding of the mechanics of a movement [5]. For instance, during gait re-education, a physical therapist might instruct a patient to “lift the knee higher.” Such cues, they argue, allow for precise adjustments and refinements. On the other hand, external focus proponents emphasize the naturalness and fluidity that come with external cues [3]. They point to the transformative effects of attentional focus in sports and dance. A golf coach might say, “Aim for the tree in the distance,” shifting the golfer’s attention from the mechanics of the swing to the desired outcome. Notably, suppose the attention is directed on the desired movement effect (i.e., external focus). In that case, it has been demonstrated that an active person produces more effective performances in sprinting maximally [6], jumping as high as possible [7], learning a specific ability like kicking a ball [8] or playing a piano [9]. Listening to music (viz., another external focus) increases heart rate without increasing the perception of effort during brisk walking [10]. Additionally, the positive results associated with an external attentional focus have also been observed in populations with various health conditions, from obesity to neuromotor impairments [11–16], lower limb prosthesis [17] or visual impairments [18].

When it comes to intellectual disabilities, there is a wide range of genetic conditions (e.g., Down syndrome, Fragile X syndrome, William’s syndrome) leading to significant impairments in intellectual and adaptive behaviour during the childhood period [19]. In particular, Down Syndrome (DS) is a common chromosomal syndrome composed of an extra copy of the human chromosome 21 that can present several disorders: cardiovascular and respiratory problems [20], joint hypermobility, low muscular strength, poor balance [21] and delayed acquisition of gross motor skills in running, walking, jumping and throwing [22]. Regarding cognitive performance, it has been shown that attention is reduced in children and adolescents with intellectual disabilities [23]. Selective attention, sustained attention and divided attention are important cognitive characteristics that are poorer in people with intellectual disabilities [24], with enormous discrepancies among each syndrome. For instance, the ability to select relevant stimuli from irrelevant ones, the ability to attend to two tasks at once, or the ability to maintain focus over time are majorly affected in children with Fragile X syndrome or Down Syndrome [25]. Learning and memory for verbal and nonverbal complex information are also compromised in children with DS [26].

Enhancing the overall well-being and quality of life of individuals with DS necessitates the improvement of their movement quality. DS is linked to various physical difficulties, such as hypotonia, joint hypermobility and diminished coordination, which can substantially affect mobility and motor abilities [27]. Individuals with DS can obtain various advantages by prioritising enhancing movement quality through specific interventions like physical therapy, occupational therapy and exercise programs. Improved movement quality enables better performance of everyday tasks, enhances autonomy and engagement in activities, and improve the efficacy of rehabilitation programs. Furthermore, enhanced movement proficiency can aid in preventing musculoskeletal disorders, mitigate the likelihood of obesity and promote cardiovascular well-being, fostering a healthier and more physically engaged way of life. In addition, improved mobility quality promotes social inclusion by allowing individuals with DS to participate more actively in recreational and social activities, resulting in increased overall enjoyment and satisfaction. Hence, it is crucial to prioritise strategies that target enhancing movement quality to optimise the physical and psychosocial well-being of individuals with DS. In this sense, modulating the verbal cue during training or teaching a specific movement is a fundamental strategy that a physical trainer or school teacher should consider using with people with DS. To the best of the authors’ knowledge, only one study confirms the usefulness of adopting an external focus on motor learning in individuals with intellectual disabilities, highlighting better performance when adopting an external rather than internal focus cue [28].

In summary, adopting an external focus of attention has been demonstrated to optimize fine motor performance and the learning process, suggesting its potential usefulness in individuals with intellectual disabilities. Therefore, our research aims to investigate the role of attentional focus on motor performance in DS individuals. As per previous relevant literature [2], we hypothesize that people with DS benefit from adopting an external rather than an internal focus to improve gross motor performance. Therefore, objectives of the present pilot study were to evaluate the feasibility of instructing DS individuals to perform few specific gross motor tasks by focusing their attention on an external rather than internal goal, and to evaluate the effect sizes of the EF approach compared to the IF one on the performance of such tasks.

## Materials and methods

### Participants

The recruitment period for this study was from 15.11.2023 until 15.12.2023. Seven adult participants (5 males, 2 females) with DS (age: 25.2±3.2 yrs, height: 1.70±0.04 m, body mass: 72.0±6.3 kg) living in community dwellings voluntarily participated in the study. The inclusion criteria were a mild intellectual disability (IQ = 51–69) defined with the Wechsler Scale of Intelligence [29], an established ability to understand basic verbal communication, independence from personnel or assistive support devices, absence of remarkable sensorial or physical impairments, behavioural problems or any other clinical condition that may compromise the practice of physical activity. After a detailed explanation of the study’s aims, risks and benefits, participants or their legal guardians signed the written informed consent module to participate. The research was approved by the University of Insubria Ethics Committee (protocol number 0119168, date 19.10.2023) and complies with the Helsinki Declaration on studies with human participants.

### Procedures

A crossover study with repeated measures design was adopted. All data was collected in a structured gym room at the same time (10:30–12:30 AM). Participants completed a familiarization session during which the researcher explained and demonstrated all gross motor skills to each participant, not considering attentional focus instructions. The intervention was conducted individually and every participant had to perform the four requested gross motor skills (i.e., running, jumping, throwing and rising-up from a chair) respecting a specific verbal cue. On the first day, each individual completed the entire sequence of movement patterns following an internal focus (IF) or an external focus (EF) instruction provided in a random order (to reduce any conditions’ order effect). In the subsequent week participants performed the same tasks following the opposite cue. The attentional focus instructions are described in Table 1. Instructions were accurately written to match the participants’ readiness fully. In detail, IF leads to focusing internally on a specific body part. In contrast, during EF the attention is directed to the desired movement effect using analogies and metaphors.

**Table 1:** Attentional focus instructions.

Gross motor skills	IF instruction	EF instruction
Run (5m sprint)	Run quickly forward you by flexing your knees at 90°. Swing your arms with elbows flexed and spine erected	Run quickly forward you like a Formula One carp
Vertical jump (CMJ)	Jump as high as you can by bending both lower limbs into a squatting position. Then extend fully your knees and ankles	Jump as high as you can like a rocket take off
Horizontal jump (SBJ)	Jump as long as you can by bending both lower limbs into a squatting position. Then extend fully your knees and ankles	Jump as far away like a frog
Forward medball throw (FMBT)	Throw the ball as far as possible until the ball touches the chest. Then extend your elbows fully and throw the ball	Throw the ball as far as possible like a cannonball
Overhead medball backward throw (OMBT)	Throw the ball backward as far as possible with your arms overhead. Flex and extend your elbows fully to throw the ball	Throw the ball behind you as far as possible like a catapult
Chair rise-up (5STS)	Stand-up and sit down as quick as possible by extending simultaneously your knees with the spine erected	Stand-up and sit down as quickly as possible bouncing like a kangaroo

IF: internal focus; EF: external focus; CMJ: countermovement jump with arm swing; SBJ: standing broad jump; 5STS: five repetition sit-to-stand.

### Gross motor skill measurements

Running performance was detected by running as fast as possible for 5 meters (5m sprint) starting from a standing position. Each participant was requested to accelerate five meters from a baseline marker until the end. Three measurements for each verbal cue were executed by recording the performance time using a timing gate system (Witty, Microgate, Bolzano, Italy).

As for the jumping outcome, each participant was asked to jump vertically as high as possible, perform a countermovement jump with arm swing (CMJ) and hop horizontally as far as possible using the standing broad jump test (SBJ). Three measurements for each condition were conducted by measuring jump height using a photoelectric system (Optojump Next System, Microgate, Bolzano, Italy) during CMJ performance [30]. Similarly, the SBJ test measured the maximal horizontal distance hopped from the take-off line to the nearest heel contact point during landing [31]. For CMJ and SBJ, 90 seconds of rest was observed between each trial.

For what concerns throwing performance, it was assessed using a 3-kg medicine ball throw (circumference 0.78 m) by throwing the ball forward like a chest pass (FMBT) or backward with upper-limbs overhead (OMBT) in a seated position [32, 33]. Three repetitions for each condition were executed respecting a 1-minute rest in between.

Lastly, the functional performance in rising-up from a chair was detected through the five repetition sit-to-stand test (5STS) consisting of executing five consecutive rises from a chair with arms crossed over the chest [33, 34]. Three repetitions were performed and a resting period of 1 minute was observed. Performance time execution was recorded using a manual stopwatch.

### Statistical analysis

The normality of data distribution was checked using the Shapiro–Wilk’s test, that confirmed the non-normality of the data. Therefore, a non-parametric approach was used. The Wilcoxon signed-rank test was used to detect possible differences between each condition (internal focus vs external focus). The non-parametric effect size (ES) *r* was calculated as the ratio between the Z-score statistics and the square root of the sample size, as suggested by Rosenthal [35] for the Wilcoxon signed-rank test:

r=ZN

with *N* the number of non-zero difference pairs. The ES was classified as small (0.1≤|*r*|<0.3), medium (0.3≤|*r*|<0.5) and large (|*r*|>0.5) [36]. Significance was set with *P*≤0.05. Data are shown as Median ±Interquartile Range.The statistical analysis was performed using GraphPad Prism (version 10.2.3 for Windows, Graphpad Software, Boston, Massachusetts USA) and Wilcoxon Signed Rank test calculator [37].

## Results

**Fig 1:**
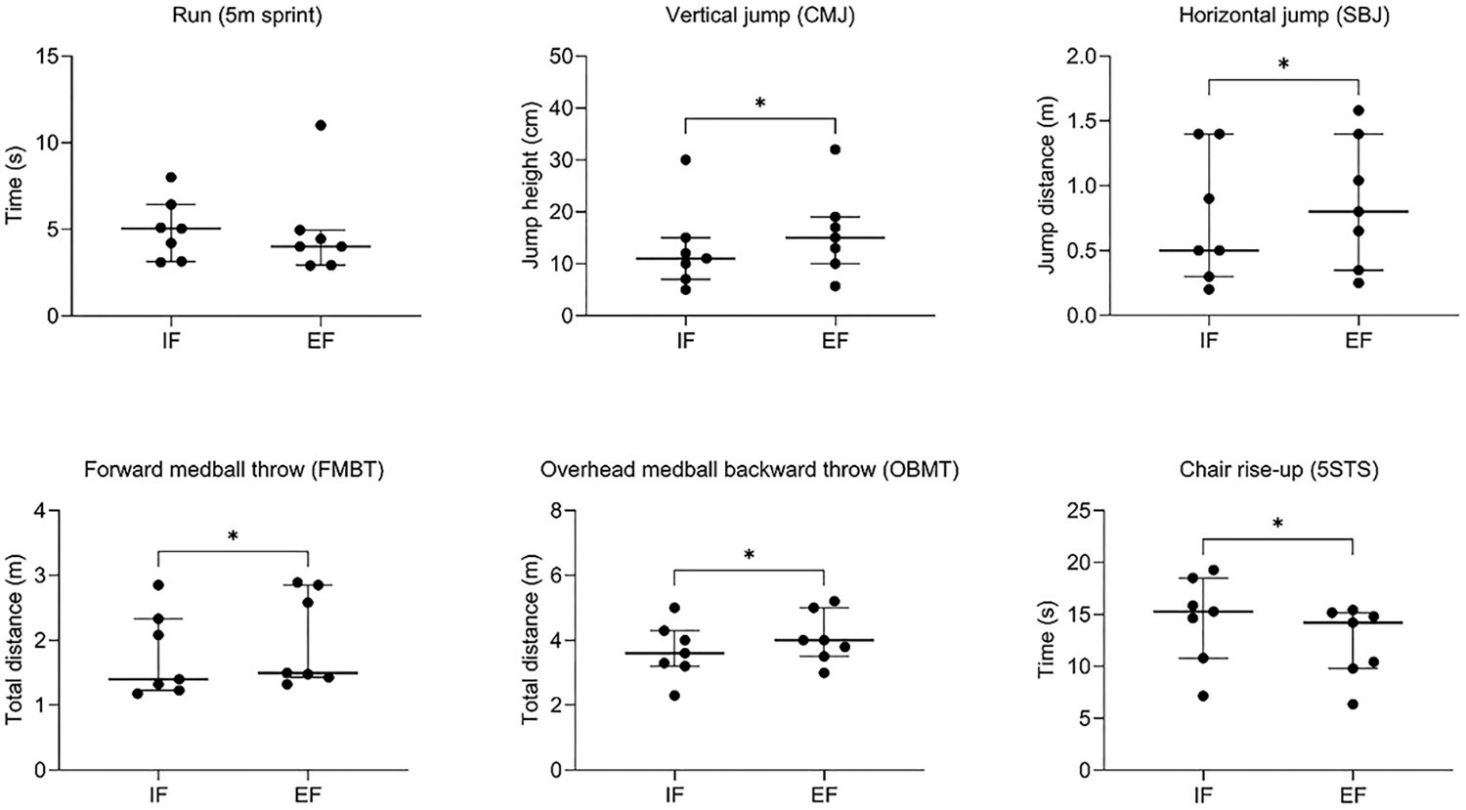
Measures of performance of different gross motor skills in IF and EF conditions. Each dot represents an individual value; the horizontal bold line indicates the median, the whiskers indicate the interquartile range.

Fig 1 graphically displays the median values ± interquartile range of IF and EF conditions in different gross motor skills performances.

The 5m sprint performance tended to be slightly better for EF (4.0±2.02 s) than IF (5.05±3.29 s) with a medium effect size (|*r*| = 0.39) but the difference between conditions did not reach the statistical significance (*P* = 0.29). By contrast, all the other measures of gross motor skills were significantly better for EF than IF, with a large effect size.

In particular, as for the jumping performance, the CMJ test showed a significant difference in favor of EF instruction (EF = 15.0±9.0 vs IF = 11.0±8.0 cm, *P* = 0.015, |*r*| = 0.91). Similarly, the SBJ was superior when adopting an EF cue (EF = 0.8±1.05 vs IF = 0.5±1.0 m, *P* = 0.031, |*r*| = 0.88). As for throwing performance, an EF instruction was better than an IF one in both FMBT (EF = 1.5±1.42 vs IF = 1.4±1.1 m, *P* = 0.015, |*r*| = 0.91) and OMBT (EF = 4.0±1.5 vs IF = 3.6±1.1 m, *P* = 0.031, |*r*| = 0.88). Finally, the 5STS performance was better adopting an EF than IF instruction (EF = 14.22±5.38 vs IF = 15.28±7.7 s, *P* = 0.015, |*r*| = 0.91).

All individual data are available in the S1 File.

## Discussion

Two were the objectives of our study, originally motivated as pilot research: evaluating the feasibility of adopting EF or IF instructions to improve gross motor skills outcomes in individuals with DS, and assessing the effect sizes of the EF approach compared to the IF one. As to the first objective, we demonstrated that it is possible to adopt a specific attentional focus cue within individuals with a mild cognitive disability who are moderately active and independent. This because our DS participants could understand basic verbal communications and discriminate the meaning among various attentional focus instructions. However, the main result of our study was to find large effect sizes in five out of six motor skills considered and this allowed us statistically demonstrating the superiority of the EF approach in all the motor tasks except one. Such a result is relevant because attention deficits and delays in motor skill development, such as running, jumping or throwing patterns, are particularly evident in DS [22, 24]. Although we did not demonstrate that the running performance for the EF approach is significantly better than the IF approach, its medium effect size further supports the use of an external focus to improve movements performance.

Jumping exhibited the highest performance in both CMJ and SBJ assessments along with an external focus instruction. Similarly, Kershner et al. [38] significantly ameliorated jump height, peak velocity and mean concentric velocity during a vertical CMJ test when using an external focus in NCAA Division I baseball able-bodied individuals. On the same line of evidence, a recent review highlighted the importance of adopting an EF to enhance motor learning during a horizontal jump [39]. A verbal instruction that privileges an external focus of attention obtained greater lower-limb force production with lower muscular activity than cues that encouraged an internal focus, reflecting a greater movement efficiency in movement production [40]. Indeed, the external cues adopted in the present study such as “Jump as high as you can like a rocket take off” or “jump far away like a frog” demonstrated the superiority of an external focus on the jumping gross motor performance, as compared to an internal focus, that requires to concentrate on the body’s movement.

When dealing with throwing outcomes, our results support the potential effects of following an external focus of attention. Nevertheless, research has only sometimes shown performance benefits with an EF when performing upper-body exercises such as bench press [41, 42] or midthigh pull [43]. When the movement complexity increases (i.e., during clean and jerk exercise), accumulating evidence suggests the potentiality to shift the focus outside of the body [44].

The significantly better performance in the chair-rising test during an EF cue was unsurprising, but it should be contextualised to the specific population. For instance, obese individuals had better gross motor performance in chair stand following an intervention based on EF cue [11]. However, when considering elderly individuals, the assumption that an external focus may be superior is still unclear [45].

The only gross motor skill in which EF did not result to be significantly better than IF was the 5m sprint. However, 6 out of the 7 participants performed better after receiving an EF instruction, with a medium effect size. To obtain a significant difference between two conditions when the effect has a medium size, a population about five times larger than the number of participants enrolled in our study is required. Thus, we cannot exclude that a positive effect of EF instructions can be revealed on the 5m sprint too in a larger future study.

A superior performance following an external cue in both able-bodied or intellectually disabled individuals should be partially explained by the *constrained action hypothesis* [3, 4, 46]. The *constrained action hypothesis* suggests that an external focus of attention permits automatic behavior, non-conscious motor control and natural movement with the result of a more fluent movement execution [3]. On the contrary, when shifting the attentional process internally, the outcome is more constrained and consciously controlled with the consequence of depressing motor performances due to a more controlled movement action [3, 46]. An EF would modify neural activation *de facto* by reducing the activation of the upper motoneurons with a slower motor pathway [47, 48]. This type of instruction may increase intramuscular and intermuscular motor system efficiency by ameliorating both motor performance and the learning process [49].

An external focus instruction might be useful for practitioners with intellectual disabilities [28]. This is particularly relevant because people with DS demonstrate poorer selective attention (i.e., the ability to focus the attention on completing the task only on relevant information), sustained attention (i.e., the ability to concentrate on the whole movement task duration) and divided attention (i.e., the ability to discriminate information by dividing the attention between listening to the educator or coach while completing the task [25]). An EF allows attention far from the body thanks to the adoption of metaphors and analogies (e.g., “explode by jumping high like a rocket”) by simplifying information, promoting a more unconstrained movement with less cognitive demand so that it might be particularly helpful for individuals with cognitive impairments. Nonetheless, it seems that integrating EF instruction during gross motor skill performance in individuals with DS allowed them to perform better in patterns with a more pronounced acquisition delay due to the nature of disability [22].

### Limitations

Our work presents four limitations that warrant attention. First, the study was designed to directly compare the EF with the IF approach on the resulting motor performance, thus baseline measures of the motor performance before receiving any external or internal instruction were not collected. For this reason, however, we cannot quantify whether and to what extent the IF approach too may have improved the motor performance. Second, the specificity of DS does not permit generalizing our results to other intellectual disabilities [50]. Third, the absence of a retention test makes it impossible to ascertain the complete efficacy of adopting an EF instruction. Fourth, the absence of neuromuscular (e.g., EMG) or physiological (e.g., heart rate monitoring) measurements limits the results’ interpretation [51].

## Conclusions

Overall, although obtained on a small group of participants, the results of our study demonstrated that an external focus of attention elicits superior gross motor performances in individuals with DS. Vertical and horizontal jumping and throwing or rising-up motor skills could be positively affected by adopting an EF instruction compared to an IF one. Physical trainers, operators in rehabilitation, or teachers should be encouraged to manage different types of attentional focus cues, especially external ones, to improve cognitive and gross motor performances in persons with intellectual disabilities.

## Supporting information

S1 File All individual data. (XLSX)
